# First-time parents' experiences of home-based postnatal care in Sweden

**DOI:** 10.3109/03009730903431809

**Published:** 2010-04-07

**Authors:** Katarina Johansson, Clara Aarts, Elisabeth Darj

**Affiliations:** ^1^Department of Women's and Children's Health, International Maternal and Child Health, Uppsala University; ^2^Department of Public Health and Caring Sciences, Uppsala University, UppsalaSweden

**Keywords:** Breast-feeding, early discharge, home-based postnatal care

## Abstract

**Aim:**

To gain a deeper understanding of first-time parents' experiences of early discharge from hospital after delivery and home-based postnatal care.

**Material and methods:**

The study was comprised of focus group interviews, interviews with couples and with fathers. Twenty-one parents participated. Inclusion criteria: healthy women who have given birth to their first child after a normal pregnancy and delivery, the women's partners, healthy and full term babies, Swedish-speaking, discharge from the delivery ward within 24 hours, resident in the Uppsala community, the parents cohabited at the time of the delivery. The material was analysed by qualitative content analysis.

**Results:**

Three themes emerged: *The family's strategy*, which describes the family's expectations of postnatal care and their experiences of the real situation. Some are flexible concerning going home early, and others have decided in advance. *Self-reliance and strength*, which explores the parents' feelings of security and uncertainty, freedom and independence, and shared responsibility. Breast-feeding is described as the ‘main thing’, an interactive learning process. *Professional support in the home* summarizes the parents' experience of the midwife's support at home. While conflicting feelings may be revealed during the first days, the midwife confirms their new roles as parents. The midwife is seen as a support and adviser to the parents.

**Conclusion:**

This study shows that parents welcome home-based postnatal care with professional support from midwives. We conclude that this care suits healthy families. We think it will be more important in the future to discriminate between healthy families and those in need of hospital care, than to focus on the moment when they leave the hospital, early or late.

## Introduction

Worldwide the home is the most common place for child-birth, and according to the World Health Organization it is not essential that healthy women and children be cared for in hospital (1). In middle- and high-income countries, however, it is more common to deliver in hospital. At the beginning of the 1900s as many children as possible were born at home in Sweden. During the 1930s postnatal care was extended and developed, and in the 1960s 99.7% of women gave birth in hospital (2). In the past 30 years the length of hospital stay after vaginal delivery has become shorter. In Sweden in the 1970s, the average length of the stay in hospital was 6 days, which gradually declined to 2 days in 2005 (3). The present study is focused on parents who left the hospital even earlier. Apprehension about early discharge after delivery has been expressed concerning a shorter breast-feeding period, increased re-hospitalization of mothers and children, women losing confidence over the care of the child, dissatisfaction with the postnatal care, or an increased risk of maternal depression (4). There have also been fears about increased severe neonatal jaundice, breast-feeding malnutrition, and about heart problems not being detected (5–7). It has been debated whether breast-feeding is affected by early discharge after birth, and it has been shown that those who left the hospital early with support at home had a higher frequency of breast-feeding or a longer breast-feeding period compared to those who stayed in hospital (8,9). Other studies, however, report no difference in the relationship between the length of time breast-feeding was given and the length of the stay in the maternity ward (10,11). In the UK, investigations on the connection between early discharge and re-hospitalization within the first month reveal that 2.8% of new-borns are re-hospitalized due to infections, ‘colic' feeding problems, and jaundice. The authors conclude re-hospitalization is not dependent on the time of leaving hospital and that breast-fed children are at lower risk of re-hospitalization (12). In Sweden, the risk of re-hospitalization was similar when comparing those cared for in the maternity ward and those who were discharged early, 1.7% (13).

Early discharge from hospital and home visits by midwives was introduced in Sweden in 1984 (14). Since the early 1990s our clinic has aimed to distinguish between healthy women and children after delivery, and women and children in need of hospital care in order to provide the most appropriate postnatal care. Healthy women with a healthy child are offered postnatal care in their own homes by the Postnatal Visiting Midwives Group (PVMG). On the other hand, unhealthy women or babies in poor health will be cared for in the hospital. In 1993 The National Board of Health and Welfare defined early discharge as the mother and child being discharged from hospital after a minimum time of 6 hours after delivery and at the latest after 3 days in the maternity ward (13). Each clinic was supposed to provide written guide-lines and ensure that quality was maintained. The National Board of Health and Welfare emphasizes that there should be a well functioning chain of care both before and after child-birth. An early discharge, say, 6 hours after delivery requires a healthy mother, a normal pregnancy and delivery without complications, a healthy baby born at full term (gestational age 37–42 weeks) with normal weight, and this baby must have been examined by a paediatrician (13). Parental support is offered to parents, and a plan is drawn up on the basis of their needs and desires as far as knowledge, information, and contact with other parents in questions about parenting and children's development are concerned (15). The purpose is to empower the parents and enable them to make use of their own capabilities and resources (16). In Sweden partners have parental leave for 10 days which is used in relation to delivery. Britton et al. show that if the first days are spent at home, the family has the opportunity to be together, and this leads to the father playing a bigger role in the care of the child (17). The economic considerations have been examined in several studies, and early discharge is considered both cost-effective and safe, provided there are home visiting activities (18,19).

In our clinic, specially trained midwives offer home visits as often as the families need until the fourth day after delivery. Families who decide to go home early are contacted by telephone the day after their arrival at home, and during the home visit a check-list is used. The check-list is presented elsewhere (8). By following this, the midwife gains information about the mother's and the child's health and about how breast-feeding is proceeding. On the fifth day, the family returns to the hospital for a second paediatric examination and a metabolic screen. The need for information during home visits has been investigated, and the results reveal that parents have most questions about nursing the new-borns (20).

PVMG in Uppsala is an example of a change in caring routines aimed at providing good care and maintaining high medical security and quality. It is important that this home visiting activity is continually evaluated. The aim of the study was to gain a deeper understanding of first-time parents' experiences of early discharge after delivery and home-based postnatal care.

## Material and methods

The study was designed as an explorative study and conducted between April and September 2005 at Uppsala University Hospital. Out of 3654 deliveries throughout the year 19% had a caesarean section, and 6% were delivered by vacuum extraction.

Inclusion criteria for participation in the study were: 1) women and men who had had their first child, 2) healthy women with a normal pregnancy and delivery, 3) healthy and full-term children, 4) Swedish-speaking couples, 5) discharged immediately from the delivery ward within 24 hours and without further post-partum care at the hospital, 6) living in Uppsala community, 7) cohabiting at the time of the delivery. Families who met the inclusion criteria were contacted by the first author and received both a written and a verbal inquiry and information on the purpose of the study and that participation was voluntary. Three focus group interviews were conducted, of which two groups comprised four women each and one group comprised three men. There were subsequent individual interviews with four men, and three interviews in which both the woman and man were interviewed at the same time. As it was more difficult to recruit men for the focus groups, interviews were conducted both individually and in pairs to obtain the men's view-points. A question guide was used in the group discussions and interviews. The interviews were carried out within the first 2 months. The guide raised questions regarding the experience of going home immediately after delivery, the experience of home visits by the midwife, whether they felt unsure about anything, what kind of questions they had, breast-feeding, and if they had received differing advice on breast-feeding from the midwives.

All interviews were audiotaped and transcribed verbatim, after which the material was analysed for qualitative manifest and latent content (21). Qualitative contents analysis is a process for identifying, coding, and categorizing patterns in data. The analysis of the material started with the first interview. Data collection ran parallel throughout the study (22). The analysis began by going back to the purpose of the investigation and question guide. Each interview was re-read several times to gain a feeling of completeness. Meaning units consistent with the study's purpose were highlighted; these were later condensed to shorten the text but retain the content. The sections were coded and grouped into main and sub-categories that reflected the main message of the interviews. The themes were discussed between the first and second author until consensus was reached. Theoretical saturation was reached after three focus group interviews and after approximately half of the other interviews, and the material obtained was considered sufficient. The first author, a midwife with experience of home-based care, had a professional pre-understanding. In order to strengthen the reliability the first author was moderator and led all the interviews in the presence of an observer who provided insight. One-third of the material was coded by one of the co-authors.

### Ethical considerations

The Research Committee of Uppsala University, Sweden approved the project. Confidentiality was guaranteed as only the authors had access to the material. An ethical dilemma is that complete anonymity can never be guaranteed when focus group interviews are used.

## Results

During the study period a total of 46 women delivered their first child and left the hospital within 24 hours after the delivery. Of these, 25 lived within the Uppsala community and fulfilled the inclusion criteria for participating in the study. Twenty-one persons were recruited for the study, 11 women and 10 men. All had had their first child except for one man, who had a child from a previous relationship and should not have been included in the study according to inclusion criteria. The women were between 25 to 34 years of age (mean age 30.1). In Sweden the average age of primiparas was 29.0 in the same period of time (23). The families left the hospital between 6 and 17 hours (mean caring time at the hospital was 10.4 hours) after the baby had been born.

**Figure 1.**
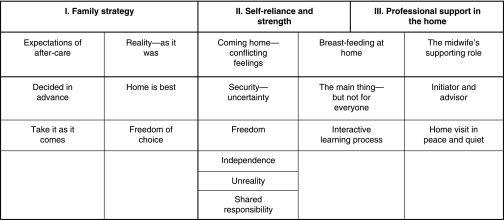
Overview of the themes: main and sub-categories.

The content analysis revealed three themes: *Family strategy*, *self-reliance and strength*, and *professional support in the home* (Figure 1). The themes reflect the latent content and to some extent overlap the underlying categories. Each theme contains the main and sub-categories expressing the manifest content. The quotes presented were chosen, as they represent the core of the category. If a quote was too long to reproduce entirely it was denoted by ‘…’.

### Family strategy

Most parents knew about the PVMG due to information given during the pregnancy at the antenatal clinic and from friends. Their parental expectations of home-based postnatal care could be divided into two categories: those who had ‘decided beforehand’ and those who wanted to ‘take it as it comes’:

We wanted to go home as early as possible. We didn't want to stay at all (man, 30).

… it was difficult to say beforehand. But, we had to see what it was like … (man, 31).

Many parents preferred to go home and rest when mother and infant were well and healthy after an uncomplicated delivery. Some expressed that ‘home is best’; they were tired, but they felt good and thought they could sleep better in their own home.

It was just that I felt good and I was so tired, and my own bed… wonderful! (woman, 34).

Approximately half of the families had planned to stay, but when they felt good, they changed their minds and accepted the offer of home-based continuation of the postnatal care. Some parents felt that the home journey involved ‘freedom of choice’ although with some modification; some parents took the initiative to go home themselves, but some were asked by the staff. One family had the impression that if everything was normal, they had no choice, and they should be sent home. This particular family, however, wanted to benefit from the postnatal care at home. Most of the parents had the feeling that they were allowed to make the choice, and they were both involved in the decision.

… they asked us if we were ready … to go home … and so we said, of course we can go home (man, 38).

… it was so good that we both thought that … we wanted to go home, both of us took responsibility, not just the mother (woman, 30).

In addition, there was ‘freedom’ to do as they liked at home.

… and in the middle of the night… you could walk around a little and sit down in the living room (man, 30).

### Self-reliance and strength

All parents pronounced that they felt good to go home, although conflicting feelings could develop. At home, families expressed that they had to rely on their own abilities. They felt ‘unsure’ with their new-born baby. At the same time they felt safe to find themselves in a familiar environment, and they knew they could get in contact with midwives easily day and night, and that a home visit was planned for the next day.

I am familiar with everything here … I am in control in the apartment … that feels very secure (man, 31).

However, some parents said they felt uncertain about everything, others articulated uncertainty especially about nursing and care of the baby, and others did not feel at all uncertain. The parents were in new circumstances and had to find the best solution as they could. This was expressed in a positive way, about freedom, self-reliance and shared responsibility, as well as independence. Later they could always ask the midwife if they had done it right. The midwife supported them in their new role and strengthened their self-reliance.

It is at home that all the questions crop up … if I had still been there, I probably wouldn't have thought in the same way … independently (woman, 34).

Besides feelings of security and uncertainty, freedom and independence, there was a feeling of ‘unreality’, as there was one more in the family.

… at the same time it is strange. In the morning I got a son and we are at home at three o'clock. We came home with three instead of two (woman, 34.)

Shared responsibility was also part of the new situation. At home the partner took care of the domestic issues, and the mother could rest and breast-feed the baby undisturbed. The parents thought that breast-feeding was important but difficult and described it as the ‘main thing’:

… breast-feeding is just so important… it is almost a bigger thing than giving birth… it is so essential that it works in some way (woman, 29).

However, this was ‘not for everyone’. Some women thought it was boring, difficult, and took too much time to breast-feed, but they knew it had to be done anyway:

Everyone says that is it is so wonderfully natural … quite honestly, I think it is extremely difficult to breast-feed (woman, 32).

Over time, it became easier and they saw it as ‘an interactive learning process’. Through breast-feeding, the women learnt to trust themselves and their children. Breast-feeding was described as an interaction between the mother, the child, the father, the midwife, and their environment. Eventually they found their own way and learned to trust the child and themselves.

… he knows himself when he is hungry… if he cries, he gets food… he stops when he is full (woman, 28).

### Professional support in the home

Care in the home was divided into the midwife as ‘initiator and advisor’ and ‘home visit in peace and quiet’. The parents thought it was good that the midwife initiated contact; this meant they did not have to get in touch themselves and explain why they wanted a home visit. The majority of parents thought that the midwives gave them consistent advice. The midwives were considered experienced and professional, and the parents felt that their new role was confirmed and the midwife gave them confidence:

If you call and ask… do you want us to come… it feels as if it is already planned… it feels welcome… not inconvenient (woman, 30).

All parents considered the home visits good and said that the midwife had time and could stay as long as she was needed.

… it was not as if she stayed a long time … nor the feeling that she wanted to leave … we got the time we needed … that felt good (woman, 26).

… it is all about having someone … to talk to, who can reassure you and make you trust yourself as a parent… that we will make it… (woman, 26).

## Discussion

The present study gives us a deeper understanding of the family 's strategies and expectations of postnatal care and how they experienced the first days. It has been previously reported that women were more positive to this kind of care if they received information during the pregnancy, as a voluntary option or they had used this kind of care before (8,22). They felt secure when the midwife was easy to reach by telephone (24). Studies report that parents who felt tired after the birth thought they rested better at home (25,26). This study, however, shows that the attitudes towards early discharge can change after the baby is born. The family strategy was to be flexible and see how things worked out, depending on the situation after the delivery. This seems practical and also shows autonomy when relying on professional support at home. Evaluating parents' feelings about going home early support this conclusion. The parents trusted their own ability and felt confident when they had as close contact as they needed with a midwife. The ability to think independently was obvious when they tested themselves at home; they could set up their own routines and their new role as a parent was confirmed by the midwife. The feelings of unity with their partner and shared responsibility have been reported in other contexts (25,26). In this study, parents emphasized it was a joint decision to go home early.

The women felt breast-feeding was a process and learnt to trust themselves and the baby. If breast-feeding worked, then everything else functioned. One woman described breast-feeding as the ‘main thing’, but there were women for whom breast-feeding was difficult, also confirmed by Kellerher in a study from USA on women's experiences of pain and discomfort while breast-feeding (27).

We learn that the midwives' initiative, contact, and offer of home visits were welcomed. Women were reluctant to call the midwife, as they did not want to disturb her (28,29). It seems important that the midwife initiating contact with the family continued to do so during the whole period. When parents felt insecure, they received confirmation from the midwife that they were doing the right thing, taking care of the new baby and breast-feeding. In this study the midwives strengthen parents' self-confidence and confirm that the parents themselves are experts on their child's care and in their own lives. Parents have the ability and willingness to take responsibility and are the key people in taking care of their child (30). Thus, the health professionals took a supporting role. In Sweden, the midwife is traditionally responsible for normal pregnancies and child-birth and thus plays an important role in supporting and strengthening what is normal and unique in child-birth (16). For the parents, the time after delivery is a transition to a new phase in life, and this study highlights that parents are positive about spending this transition time in their own home (28).

All, but one, of the participants shared the common experience of having their first child, and this homogeneity may have made it easier for them to share their experience (31). To be able to hear the men's view-points, individual and pair interviews were used. Some researchers recommend this triangulation method in order to obtain as much information as possible (32,33). The majority of parents were positive about the care they received after delivery, and it is possible that those who were negative about the care declined to participate.

A homogeneous sample of healthy families was studied. They had all had a normal and relatively quick delivery. The results are not transferable to a larger group but can describe experiences among families in the same situation. The results of this study indicate that home-based postnatal care suits first-time parents and empowers them in early parenthood, given that an experienced midwife provides professional support. An alternative to the PVMG could be that the responsible antenatal midwife, who meets the women and men throughout the pregnancy, will also perform the home visits after birth, providing a unique continuity in care. In addition, clinical infections with antibiotic-resistant bacteria, such as Metillicinrestistant Staphylococcus areus (MRSA) and *Klebsiella* Extended Spectrum Beta Lactamase (ESBL), is a growing threat to hospitalized patients and especially to neonates, with high mortality among them (34). Recently three new-borns died due to untreatable *Klebsiella* ESBL infections in a Swedish hospital (personal communication). It will be even more important in the future to decrease the number of healthy people being hospitalized and exposed to untreatable infections. Healthy mothers and children will certainly have to go home early, and our responsibility is to provide a well organized health care system to support the parents without risking patients' security. Furthermore, planning the building of new hospitals will focus on wards with exclusively single rooms in order to diminish the risk of contracting nosocomial infections, and recommended standards for planning new-born intensive care units have been presented (35). In this study we have gained information from parents who have experienced home-based postnatal care, and we can recommend it. We think, however, that it is essential to discriminate between healthy families and those in need of hospital care, instead of focusing on the time when the parents left the hospital after the delivery.
